# Supplementary material for: MAD-FC: A fold change visualization with readability, proportionality, and symmetry
Source: PLoS One. 2024 May 31;19(5):e0304632. doi: 10.1371/journal.pone.0304632 (PMC11142613; doi:10.1371/journal.pone.0304632)
Supplement: Attachment — Submitted filename: CommentsToAuthor.pdf [file pone.0304632.s001.pdf]

Review of Corliss *et al*., 2023 (“MAD-FC: a fold change visualization with readability, proportionality, and symmetry”)

Corliss et al. describe MAD-FC, a transform for fold change values designed to produce easier-to-interpret visualizations (compared to visualizations of raw fold changes and visualizations of log fold changes). The authors outline four desirable properties of fold change visualizations (readability, proportionality, symmetry, and high dynamic range), argue that plots using MAD-FC-transformed fold changes exhibit readability, proportionality, and symmetry, and present various examples of these plots compared with plots of raw and log fold changes.

Visualizations of raw fold changes do not represent negative fold changes well: although positive fold changes fill the interval of $(1, \infty)$, negative fold changes are constrained to the interval $[0, 1)$. The scale of fold changes in a MAD-FC plot is adjusted to account for this, allowing for negative and positive fold changes of equal magnitude to be symmetrically placed on both sides of 1 (the point marking “no change”). Log plots of fold changes also exhibit symmetry, since $\log(A/B) = -\log(B/A)$ for all positive A and B; however, unlike log plots, MAD-FC plots preserve fold changes’ linearity.

I believe that the proposed methods have merit and could be widely useful. However, the paper has some serious issues that should be addressed before publication. Among other major and minor criteria, some important issues I see include:

- I am not convinced that the four described properties of fold change visualizations are well-defined;
- The methods detailing the transform are not clearly written;
- The paper lacks details describing how Figures 3–7 were created, and does not provide information about the locations of the corresponding data;
- The paper uses inconsistent terminology and has a nontrivial amount of typographic errors; and
- I believe that the paper places too much of an emphasis on axis ticks and labels rather than on the MAD-FC transform itself.

Below are my comments. Please note that I will refer to page numbers in the draft PDF (spanning 43 pages): specifically, I will refer to pages in the first version of the manuscript within this PDF (within the range of pages 7–28).

Major points

1. The words “graph,” “plot,” and “chart” are all used (apparently interchangeably) throughout the manuscript (for example, the abstract says “plots of fold change” and “fold change charts” in multiple places).

The third reference that the authors cited (Midway 2020) agrees that these terms are often used interchangeably (see Box 1 of that paper). For the sake of clarity, I recommend sticking to only one of these terms throughout the manuscript, since the definition of “chart” given in the Background section (page 9) makes it seem as if charts are distinct, somehow, from plots and graphs. In particular, I suggest replacing “graph” and “chart” with “plot” (since by my count “graph” is used 4 times in the paper, “chart” is used 5 times, and “plot” is used around a hundred times), although I leave the decision up to the authors.

2. Many parts of the paper refer to “log plots” and “linear plots” of fold change, and a recurring implicit claim in the paper (including at least the title, abstract, and introduction) is that MAD-FC represents a new visualization method, in addition to a new mathematical transform.

   I contend that referring to MAD-FC in this way overcomplicates the paper, and that it would be clearer to just refer to MAD-FC as a transform (albeit a transform which has been defined primarily for the purposes of visualization). This is analogous to how we can define a logarithmic transform, but the logarithm (by itself) is not a visualization method: it is a way we can adjust the scale(s) being used in a visualization. We might sometimes refer to “log plots” in shorthand, but these are more precisely described as “plots of log-transformed data.”

   Similarly, the authors use the MAD-FC transform in multiple types of “MAD-FC plots”—in ordinary scatter plots (Figure 1F, 2E–G), volcano plots (Figure 3C), box plots (Figure 5C), heatmaps (Figure 7C), etc. It’s subtle (and I understand if the authors have reservations about making this change throughout the entire paper), but I strongly recommend adjusting the paper to move away from the term “MAD-FC plot” in favor of describing the “MAD-FC transform,” the results of which can then be visualized in many different types of plots. (Another fix would be explicitly defining “MAD-FC plot” to mean “any plot that uses MAD-FC-transformed fold changes,” etc.).

   When I first began reading through this paper, I assumed that the authors were defining a new type of plot—but the main contribution here (and it is a worthwhile contribution!) is the transform that the authors present, rather than the way this transform is plotted. This is what is worth emphasizing.

3. I propose replacing the term “reference point” with “point of no change,” or something similar. This will make it consistent with the term “fold change from no change” (also, the terms “reference point of no change” and “point of no change” pop up multiple times in the paper anyway).

4. The paper introduces the term “fold change from no change”, but also makes use of the term “fold change units” throughout. It seems to me like these are different ways of describing the

same concept, so I recommend sticking to one of them. (If I'm reading this wrong and they are two separate things, that should be made clearer—I don't see much reference information online when I search for "fold change units", so I expect other readers will be similarly confused.)

5. I think the definitions of "readability" (Background, page 10: "a visualization with readability has a clear and direct mapping between the value of the datapoints and their spatial location") and "proportionality" (Background, page 10: "a visualization exhibits proportionality if the fold change datapoints going the same direction are proportionally distant from the value denoting no change within the plot") need some work. I suspect that it might be best to merge "readability" and "proportionality" together, since these terms' definitions seem to overlap.
    1. Arguably, a visualization using a logarithmic scale also has such a clear and direct mapping—the only difference is that it is a logarithmic mapping, and thus can be slightly harder to interpret. You may want to consider renaming "readability" to "linearity," or saying "a visualization with readability has a [linear] mapping…", or something similar.

    2. In the sentence on page 10 that says "A log plot would be considered partially readable by our definition- while the axis labels reflect the range of the dataset": I am not sure what the authors mean by "the axis labels reflect the range of the dataset." I'm having a hard time imagining a plot in which this is not the case (the only situation I can think of is if you truncate the axis of a plot and thus leave out some datapoints).

    3. The section on "readability" describes this property in terms of axis labels and ticks, but I propose that this section would be clearer if phrased in terms solely of the scale in use. This is because the choice of what ticks to draw on an axis (and what to label these ticks) is independent of the underlying scale. For example, you could imagine a linear scale of fold changes that only has ticks at 1/10, 1, and 10; or you could imagine a log plot where the axis labels are extended to show both the log fold change and the corresponding raw fold change (e.g. something like "2^0 = 1", "2^1 = 2", etc., which would combine Figures 1D and 1E into a single plot).

    4. I think the section on "proportionality" should be adjusted to be more explicit that a visualization "exhibits proportionality" if fold changes' distances from the point of no change are proportional to the magnitudes of their fold change from no change. The current wording is a bit confusing.

    5. From page 10–11: "This property allows for direct comparisons between the magnitude of positive fold changes separately from negative fold changes."—the meaning of this sentence is unclear to me. I'm struggling to see how this is meaningfully different from the "readability" property. Since "readability" also references proportionality ("A linear

visualization exhibits readability because […] the precise value of a datapoint is in proportion to its distance to the nearest axis labels”), you may want to consider merging “readability” and “proportionality” into a single property called “proportionality to fold change from no change” or “linearity” or something.

6. From page 11: “This property makes it difficult to compare the magnitude of fold change datapoints based on their distance to the reference point.”—this is a strong claim that I’m not sure I completely agree with. As the magnitude of a fold change increases, the magnitude of the corresponding log fold change (aka the distance to the reference point in these plots) also monotonically increases, so we can still technically compare any two log fold changes based on their distances to the reference point. I recommend adjusting this claim slightly, to be clearer that the problem is that it can be harder to compare values on a log scale than on a linear scale.

6. I think the definition of “high dynamic range” (Background, page 11) also needs improvement.
   1. According to the Abstract, page 8, a visualization with high dynamic range is one where “datapoint values are discernable across orders of magnitude”. The exact meaning of “discernable” is ambiguous here, but assuming we replace it with “distinguishable” (I think this is your intent) then we see that linear plots also technically have high dynamic range—fold changes of 0.5, 2, 20, and 2,000 are all distinguishable in linear and log plots (the difference being that in a linear plot you’ll necessarily need more space to show this data).

   2. I like the idea of relating “dynamic range” to a visualization in this way, but I think it should be approached differently (since, as discussed above, the current definition isn’t sufficient for explaining why log plots have high dynamic range but linear plots don’t). Maybe you could rephrase “high dynamic range” as the ability to show a large span of fold change values in an image of fixed size? That’s not a perfect definition, because you could always just scale down the image to fit within the fixed size, but I think it brings you closer to your intent.

   3. Lines 105–107: As with the “readability” section, I really recommend divorcing this section from the notion of “[units] on the axis scale”—it is perfectly valid to have a plot using a logarithmic scale that e.g. includes ticks that do not perfectly match the logarithmic base (e.g. 1, 2, 3, 4, 5, 10, 15, 20, … along a log10 scale). The assertion that “A log plot has high dynamic range because each unit on the axis scale represents an exponential increase of magnitude” is thus not always true. Log plots do have high dynamic range, but this is due to how logarithms work rather than how the axis is labeled.

   4. Lines 105–108: “A log plot has high dynamic range because each unit on the axis scale represents an exponential increase of magnitude, allowing scientists to differentiate fold

    changes both close in value and across a large range of magnitudes (Fig 1D).”—Focusing on the second part of this sentence: I agree that log plots help distinguish fold changes across a large range of magnitudes, but I think you could make the case that (in some situations) log plots make it challenging to distinguish similar values. For example, a plot using a log2 scale makes it easy to see that 2,000 > 2, but it might be challenging to quickly compare 1,955 and 1,960. (I’m not sure if it’s worth adding a new property to explain this or just briefly mentioning it; I’ll leave the decision up to the authors.)

    5. Lines 108–109: “A linear plot has much lower dynamic range, and typically can only differentiate datapoint values across a couple of orders of magnitude (Fig 1C).”—I do not see how Figure 1C supports this assertion. Although the negative fold changes are somewhat squished in Figure 1C, the positive fold changes can all be easily distinguished.

    6. Although the paper indicates that MAD-FC plots do not have high dynamic range, this is not explained or illustrated: the term “dynamic range” is not mentioned at all in the Methods or Results sections.
        1. Since the authors are proposing a new visualization method (which will require future users to interpret these plots, decide if MAD-FC plots will be helpful in a given situation, etc.), I strongly suggest that the authors explain the low dynamic range of MAD-FC plots in more detail.

        2. None of the examples of MAD-FC plots (Figures 3–7) is a “bad example” (i.e. the authors have made the case for all of these figures that MAD-FC plots are more useful than log and linear plots). I think it would be useful to give an example of a situation where log plots are more useful than MAD-FC plots due to the higher dynamic range of log plots. (And if such a situation does not exist and MAD-FC plots are always better, then that is worth mentioning—but I don’t think that’s the case, based on the paper.)

7. The distinction between the introduction and background sections seems slightly arbitrary, and the introduction is very short. Unless there’s a need to have a specific “background” section for PLOS ONE (I don’t think this is the case, judging by the papers I see featured currently on the journal’s front page), I propose that the authors merge the introduction and background sections into a single “introduction” section.

8. Methods, page 12: the description of “defining symmetry” is confusing. I see three possibilities; (1) are you introducing new fold change points that were not present in the dataset before, (2) are you searching through the n fold-change measurements and finding pairs that have different directions but the same magnitude, or (3) are you doing none of these things and just starting by applying the mirror transform f_M? It is not clear which of these is the case.

1. I assume almost all real datasets will be "asymmetric"—i.e. there is no guarantee that a given fold-change measurement of C can be paired with another measurement of 1/C in the same dataset. However, the datasets shown in Figure 1 and Figure 2 seem to be completely symmetric. I propose adding or removing a few datapoints in Figure 2 in order to make this situation clearer.

2. If the input dataset of fold changes does actually need to be "symmetric", then this should be clearly stated earlier; it would also represent a major limitation of the method. Fortunately I don't think this is the case, judging by e.g. Figure 3C.

3. Assuming that MAD-FC doesn't actually require a symmetric input dataset, and that it doesn't actually explicitly search for pairs of symmetric fold changes in the dataset (i.e. situation (3) as detailed above), I think that most of the first paragraph of the Methods section (and some of the next paragraph) should be removed.

9. Figures 3, 4, 5, 6, 7: Much more information is required to explain how these figures were produced. This information could go into "Methods", supporting information, or somewhere else—but the main thing is providing it.
   1. For Figure 3, for example:
      1. Where exactly did you get these data from? (The reader shouldn't need to read the other paper in order to know where the data are, what accession numbers they have, etc.)
      2. Did you begin your analysis with the raw data (e.g. reads), or from an existing count table of genes vs. samples, or from the existing output of a tool like DESeq2, or something else?
      3. How did you process the data before creating these plots? (For example, did you filter out any genes [or samples, or both] before constructing these plots?)
      4. Where did these p-values come from?
      5. What do the "Change" colors (up, none, down) mean?
   2. For Figures 5 and 6, "datasets acquired from the Cancer Genome Atlas" is insufficient—please cite the study and/or accession numbers for these datasets.
   3. Some of this information is recoverable from reading through the source code, but the reader shouldn't need to do this. In particular, the paper's Data Availability Statement should describe where the data are located; right now it just says "Yes - all data are fully available without restriction", which is insufficient.

10. Where reasonable, packages you use in your source code should be mentioned in the text, and their version numbers and corresponding publications (if applicable) should be cited. The lines

on this are a bit blurry, but I recommend erring on the side of citing things more. See PLOS ONE's Statistical Reporting section (https://journals.plos.org/plosone/s/submission-guidelines#loc-statistical-reporting).

1. For example, the source code for creating the Figure 3 volcano plots (https://github.com/bacorliss/mirrored_axis_distortion/blob/main/mirror_fc/F3A_volcano_plot2.R) acknowledges use of the EnhancedVolcano R package (https://bioconductor.org/packages/devel/bioc/vignettes/EnhancedVolcano/inst/doc/EnhancedVolcano.html), but this is not mentioned at all in the text. Please cite this package; based on the bioconductor.org link, it seems like the following citation is preferred:

   "Blighe, K, S Rana, and M Lewis. 2018. "EnhancedVolcano: Publication-ready volcano plots with enhanced colouring and labeling." https://github.com/kevinblighe/EnhancedVolcano.

2. Another example: the source code for creating Figure 5 (https://github.com/bacorliss/mirrored_axis_distortion/blob/main/mirror_fc/F5A_boxplot_gene.R) uses ggpubr, which can be cited as shown at https://rpkgs.datanovia.com/ggpubr/authors.html#citation.

11. Figure 7:
    1. It's impossible to interpret this figure as is without reading through reference 8. The reader does not know what the samples mean, what the y-axis means (I assume these are genes?), or what the expected patterns are. Please provide more information.
    2. To help resolve this problem, I propose reusing datasets between some of your figures—e.g. using the same dataset for Figures 3 and 7. If we can see the volcano plots for these data, this will help us evaluate the quality of the corresponding heatmaps.

Minor points

1. Abstract, page 8: "A useful fold change visualization can exhibit" seems like imprecise language—it could be replaced with "A fold change visualization should ideally exhibit" (or something in a similar vein). The current wording "can exhibit") implies that a useful fold change visualization could *not* exhibit any of these four properties, and I do not think this matches the authors' intent.

2. Abstract, page 8: The description of "symmetry" should be slightly expanded to emphasize that "positive and negative fold changes are equidistant to the point of no change" only applies for positive and negative fold changes of the same magnitude, e.g. +3 and -3. (The meaning is slightly ambiguous, since e.g. +3 and -1 should not be equidistant from the point of no change; this should already be clear to most readers, and this is explicitly stated later in the paper

[Background, page 11], but I can see some potential for confusion here.)

3. Abstract, page 8: This is minor, but I believe “discernable” should be replaced with the standard spelling of “discernible” (as discussed e.g. in https://archive.nytimes.com/afterdeadline.blogs.nytimes.com/2010/01/19/spelling-check/).

4. Abstract, page 8: “negative direction fold changes are bound between [0, 1] while positive are between [1, ∞]” -- I believe it would make more sense to replace the intervals here with [0, 1) and (1, ∞), respectively. This is because (i) a fold change of 1 is not a positive- or negative-direction fold change, at least as far as I can tell, so it should not be included in either of these intervals; and (ii) as I understand, infinity should not be “included” in interval notation (see e.g. https://math.libretexts.org/Bookshelves/Precalculus/Corequisite_Companion_to_Precalculus_(Freidenreich)/4%3A_Inequalities/4.02%3A_Interval_Notation#section_2).
    1. Please note that this also applies to the intervals given in Background, page 11; in the Figure 2 caption, page 12; and in Discussion, page 19.

5. Abstract, page 8: As I understand it, “applications that require a limited dynamic range” would be better phrased as “applications that do not require a high dynamic range” or something similar (followed by rephrasing the order-of-magnitude / log2-unit limits). When the fold changes you’re working with are relatively small, the visualization of these fold changes doesn’t strictly require you to use a limited dynamic range, although it is the most natural way to visualize them.

6. Introduction, page 9: “with a log plot or a linear plot (that directly presents raw fold change values)”—the authors should rephrase the sentence to clarify that the parenthetical of directly presenting raw FC values only applies to linear plots (if I’m indeed reading this correctly). Currently, the sentence is ambiguous; something like “(the latter of which directly presents…)” would help improve this sentence, I think.

7. Introduction, page 9: “Providing a novel fold change visualization may enhance the understanding and interpretation of fold change data in scientific research.” -- I recommend slightly rewording the start of this sentence to make it clearer that, in this paper, you are providing such a novel visualization (MAD-FC). For example, “[Our proposed] novel fold change visualization …”

8. Background, page 9: “Charts are visual representations used to reveal patterns, trends, and relationships in datasets (3). They cater to a wide range of data analysis goals and datatypes, and as a result, exhibit a wide range of characteristics and properties.”

1. First, as described above, I recommend replacing “chart” with “plot” here.

2. Second, I think that “patterns, trends, and relationships” are good terms to mention here, but they do not form a comprehensive description of everything in a dataset that charts/plots can be used for studying (of these three terms, only “relationships” are explicitly discussed much in the Midway 2020 reference given with this sentence). I’d recommend rephrasing this somehow to mention that these are just some of the attributes of datasets that charts can be useful for studying (but first see my comment below).

3. Third, these two sentences do not add much to the paper; they seem obvious enough to almost go without saying, especially if—as the abstract and introduction seem to assume—the reader is at least somewhat familiar with the field of bioinformatics and with the distinction between logarithmic and linear plots. I thus recommend merging these two sentences together (still citing Midway 2020) into a single sentence that (briefly) outlines that there are many different types of plots, each with their own use-cases and pros and cons.

4. Note that items #1 and #2 above both also apply to the first two sentences of the Discussion section (page 19).

9. Background, page 9: “We define four properties” → “We define four [such] properties”, or “We define four properties of charts,” etc. -- it would be good to in some way indicate explicitly that you are defining properties of plots and not of datasets. (Again, this is probably clear to most readers, but I believe being explicit here will help reduce confusion among the remaining readers.)

10. Background, page 9: “For defining these characteristics”—the use of “defining” here is slightly incorrect, because the authors already explicitly “defined” these properties in the previous sentence. I’d recommend using a different verb than “defining” (“explaining”, maybe?)

11. Background, page 9: “For a log plot, the reference point is zero, while it has a value of 1 for a linear plot.” This sentence is awkward because:
    1. The meaning of the word “it” is slightly unclear.

    2. The use of “while” is unnecessary.

    3. I would recommend being consistent with how you write out numbers (e.g. “zero” vs. “0”). (For context, see https://www.aje.com/arc/editing-tip-using-numbers-scientific-

manuscripts/.) I don't know if PLOS ONE has style guidelines for this, but if not then I don't mind as long as the authors choose something consistent (within this sentence, either saying "0" and "1", or "zero" and "one").

4. Based on these (all very minor!) items, I'd recommend rewording this sentence as something like "For a log plot, the reference point is zero; for a linear plot, the reference point is one."

12. Background, pages 9–10: I like the idea of "fold change from no change," but it was unclear to me at first how this encoding would actually be applied in practice. I would recommend providing the piecewise definition of this function in the paper (either in the background or methods sections, probably).
    1. It seems to me like this definition would be:

$$f(x) = \begin{cases} x - 1 & x \geq 1 \\ 1 - \frac{1}{x} & x \in (0, 1) \\ \text{undefined} & \text{otherwise} \end{cases}$$

       … but I'm just guessing based on figure 1B.

    2. "… that simply converts fold change measurements to the number of fold changes from zero"—I think "zero" is incorrect here, since the "reference point" for linear fold changes is one (as was described earlier in this paragraph). Maybe you could say "the number of fold changes from the reference point", if I'm interpreting this correctly?

    3. I apologize if this seems too strict; I really recommend being explicit here in order to avoid losing readers.

13. Background, pages 9–10: The current way this first paragraph is written is a bit redundant, because it introduces the "useful four properties" twice (once in sentence 3 on line 55, and again in the final sentence on line 62). There are multiple ways of adjusting this paragraph to fix this: one simple method may be replacing the final sentence with something that more clearly just says "here we explain our aforementioned four properties of a useful fold change visualization:" or something like that.

14. Background, page 10: “While datapoint values from a linear visualization is easily read”—minor grammar error: the “is” should be replaced with “are”.

15. Background, page 11 (paragraph on “Symmetry”):

    1. Line 97: I believe saying “regardless of their direction of change” is redundant. (That being said I can see how it might slightly improve readability to leave it in, so I understand if the authors would prefer to keep it.)
    2. Line 97: “allows” is a strong word to use here—the magnitudes of positive and negative fold changes can still be compared in asymmetric plots, albeit with some difficulty. I would recommend using a fairer word (e.g. “helps”).
    3. Line 98: “the magnitude between positive and negative fold changes”—I think it would be more grammatically correct to say “the [magnitudes of] positive and negative fold changes”.
    4. Lines 100–101: I think you can just refer to Figure 1C here—there’s no need to say “grey boxes to right of graph…”, since the Figure 1 caption makes the meaning of these boxes clear.
    5. Line 102: This is a small quibble, but I’d suggest writing “a two-fold change and a half fold change” differently (I don’t think the terminology “half fold change” is commonly used—plus, lines 61–62 say “a fold change of 2” and “a fold change of ½” instead). Maybe “(i.e. [fold changes of 2 and of ½] are both equidistant to zero)”?
    6. Lines 102–103: See comment about the reference to Figure 1C in lines 100–101; it also applies to the reference to Figure 1E here.

16. Background, page 11 (paragraph on “Dynamic Range”):

    1. Line 104: I’d recommend labeling this paragraph as “High Dynamic Range” rather than just “Dynamic Range”, since “high dynamic range” is the desired property of a fold change visualization you are describing. (This is analogous to how you’ve labeled the previous paragraph as “Symmetry” rather than “Symmetry and asymmetry”.)
    2. Line 104: “span across” → “span”

17. Background, page 11: “...that exhibits the readability of a linear plot (Fig 1G), proportionality (grey line overlapping with all fold change points), and symmetry (symmetrically spaced grey rectangles to the right of graph).”—as with the references in the paragraph on “Symmetry” to Figures 1C and 1E, I think this text would flow more naturally by just saying “...that exhibits readability, proportionality, and symmetry (Figs. 1F–G).”

18. Background, pages 11–12: “Similar to a log plot, a transform is applied to the raw data and then the axis tick labels are then reassigned to ensure readability (Fig 1F).”—I think you can delete

this sentence, or move its information to the caption of Figure 1 (if any of it isn't already there). The details of the visualization are out of place here, and take attention away from the previous sentence (which is essentially the thesis of this paper!).

19. Figure 1:

    1. Line 65: The abbreviation of fold change as FC here is inconsistently used, since the rest of this paragraph uses both "fold change" and "FC" (e.g. the sentence from lines 67–68 uses both wordings). Since it looks like most of the rest of the paper just says "fold change", I propose removing this abbreviation in this caption and just consistently writing out "fold change" in its entirety throughout the paper.

    2. Line 66: "illustration of the mapping between fold change values and fold change units from no change."—I recommend avoiding the term "illustration" here, since in the context of research papers that term usually refers to a picture / diagram / etc. and Figure 1B is a table.

    3. Lines 67–68: "A fold change dataset is visualized with various charts to illustrate their characteristics, with FC values ranging from 1/6 to 6."—The wording here is a bit awkward; you could say something like "A dataset of fold changes ranging from 1/6 to 6 is visualized with…" to shorten the sentence and make it easier to read.

    4. Line 69: The description here says just "symmetry", while the other uses of "symmetry" in this caption (for log plots, MAD-FC plots, and the grey rectangles) say "symmetry about zero" or "symmetry about reference point of no change". I recommend making this consistent (my vote is just making them all say "symmetry").

    5. Line 69: I'm not clear what the meaning of "standard" is here—if the intended meaning is that log2 plots of FC data are often used in the literature, then you should explicitly say that and give a reference here (e.g. the Love et al. 2014 paper that you cited as reference 2).

    6. Lines 69–72: I recommend merging some of the descriptions of Figures 1D and 1E (since the symmetry / proportionality information given for 1E also applies to 1D). Maybe swapping the labels of Figures 1D and 1E (and also swapping the labels of Figures 1F and 1G) might help, because then you can describe plots in a more natural order?

        1. This also applies to lines 72–74 for Figures 1F and 1G.

    7. Lines 70–71: "with y axis ticks relabeled to aid with reading the pre-transformed value for each datapoint"—this is a vague description, because it does not actually explain how the ticks have been relabeled. I recommend more directly explaining what has been changed in

this plot (e.g. “with the y axis relabeled in terms of raw fold changes” or something like that).

    1. This also applies to lines 72–73 for Figure 1F.

8. Line 71: the phrase “raw log2 transform values for fold change” is confusing and likely unnecessary; I recommend removing it.

9. Lines 71–72: most of the text for Figure 1E (“showing symmetry about zero, but lacking proportionality due to the nonlinear transform”) should be rephrased to make clear that this also applies to Figure 1D, since Figures 1D and 1E are identical with the only exception of different y-axis labels. Maybe move this text to the description of Figure 1D, and then adjust the description of Figure 1E to say that it’s a modified version of Figure 1D? This should make things clearer.

10. Line 74: “(B, E, G) grey line illustrates proportionality…”
    1. This is an error: the grey line is present in Figure 1C, not Figure 1B. I think this should say “(C, E, G)” instead.
    2. Also, I propose moving this information to the start of the figure caption (since multiple figures are already described at once on line 67 in “(C-G)”). But this is up to the authors.

11. In Figure 1A: The table’s column headers are “FC Linear Plot”, “FC Log Plot”, and “FC MAD Plot,” whereas the rest of the paper describes the latter type of plot as “MAD-FC plot” or “MAD plot”. (The vertical labels for Figures 1D and 1F also say “Log FC plot” and “MAD FC plot”, contradicting the column headers in Table 1A.)
    1. I recommend using consistent and concise terminology, wherever possible—both within this Figure and throughout the paper. Since the rest of the paper tends to say “log plot” and “linear plot”, one option you may want to go with is just consistently saying “MAD plot”. This would pair well the suggestion above (in “Major points”) to refocus the paper’s claims on how MAD is a new transform, rather than a new visualization.
    2. Note that Figure 3’s caption suffers from a similar problem—it writes out “fold change”, which could be confusing because the name of this method was defined as “MAD-FC”. Again, I think just naming the transform “MAD” (analogous to “log” or “linear”) would help here.

12. In Figures 1C, 1E, and 1G: I think I understand the purpose of the grey diagonal lines in these figures, but these lines are not defined consistently: the exact meaning of “grey line illustrates proportionality between each visualization and fold change units” is ambiguous, because the meaning of “proportionality with a visualization” is not clearly defined. This

ambiguity is a problem, since the lack of fit between these grey lines and the scatterplot points in Figures 1C and 1E is the rationale behind the "proportionality" rule.

1. As I understand it from looking at the provided R code (https://github.com/bacorliss/mirrored_axis_distortion/blob/cfe8e48f99475eaf155b10936e25a6f404a70673/mirror_fc/F1_transform_comaprison.R#L66 -- thank you to the authors for making this available!), it seems like in Figure 1E the grey line's slope is hard-coded to go between the leftmost and rightmost point in the plot. However, in Figure 1B, this is not the case—the line there is only aligned with the right "side" of the plot.

2. I suggest updating the Figure 1 caption (and maybe the Figure itself) to either (1) define the line in a consistent way across these figures or (2) be clearer about the exact meaning of the grey line. For (1), perhaps explicitly setting the grey as y = x (or, for Figure 1B, y = x + 1) would help? This should only visually impact Figure 1E, I think, but it would make the meaning of the grey line much clearer to the reader (while retaining the overall message of "the linear and log fold changes do not vary linearly with fold change from no change").

13. The vertical labels for Figures 1D and 1F are helpful—if isn't too much trouble, it might make this figure clearer to put one just to the left of Figure 1C saying "Linear FC plot" or something like that, to make it clearer that each row of Figure 1 describes a different type of plot (corresponding to the columns in Figure 1A).

14. As mentioned above in "Major points" for readability and proportionality, I am not sure that the step of reassigning axis labels is extremely important. It's not necessary, but you could probably combine Figures 1D and 1E into a single sub-Figure (same with Figures 1F and 1G) by using more detailed axis labels (e.g. giving both the MAD-FC and Con-MFC value for each tick).

20. Methods, page 12: It looks like "MAD-FC transform" is the only subsection heading in "Methods"—I thus propose removing this heading. (I can't tell from the PDF if "Data and material availability" is also a subsection of Methods, or if it's its own section—in any case, I think the sentence with the GitHub link could be just left as a sentence at the end of Methods. But it's not a big deal either way.)

21. Methods: Some of the inline equations are not italicized where they should be (e.g. the "x"s in line 123 are not italicized, although the "n" is italicized in line 118). If it's simple to fix this I suggest doing so, but it's not high-priority since I assume the journal will fix this during typesetting.

22. Methods, page 13, Equation (2): This equation has a special "undefined" case for fold changes that are located in the range [-1, 1), but the rest of the equations don't bother with accounting for these "undefined" cases.

    1. I recommend making this consistent, or at least adding a note somewhere in the paper that you assume that your input fold change values are positive real numbers (see the note on lines 118–119 below). If any of the fold changes are ≤ 0, this will break things (although I don't think that should ever happen in practice).

23. Methods, page 12, lines 118–119: I think the first sentence of this paragraph could just be trimmed down to "We start with a fold change dataset with n measurements." or even "We start with a dataset of n fold change values."

    1. Justification 1: there isn't a need to explicitly define these measurements as X1, X2, … Xn, since I don't believe anywhere else in the paper reuses this definition (e.g. iterating over all i ∈ [1, n]).
    2. Justification 2: I think "we wish to produce a visualization with readability, proportionality, and symmetry" should be moved into the following sentence: something like "We start with a fold change dataset with n measurements. To produce a visualization of these fold changes with readability, proportionality, and symmetry, we will perform two transforms …" would flow better.
    3. While we're at it, it would be a good idea to add some restrictions to these measurements here—e.g. stating that all n measurements must be positive real numbers (as discussed above: see "Methods, page 13, Equation (2)").

24. Methods, page 12, line 120: again, I don't think it's a good idea to so closely tie the methods of this paper with axis tick labels—I propose just removing "then reverse the transforms on the axis tick labels" from this sentence entirely.

25. Methods, page 13, line 140: "Based on this table"—what table? I can guess that this line is referring to Figure 2A, but it's not clear.

26. Methods, page 13, line 147: "a discontinuous region between [-1,1] where no fold change values can exist"—the [-1, 1] interval notation here contradicts Figure 2, which shows that one datapoint (the one colored blue, corresponding to a "no change" fold change of 1) has a MFC value of 1. I think you should instead write out the interval as [-1, 1)? (I don't think there can ever be a MFC value of exactly -1 based on the definitions given, but that is something worth thinking about.)

27. Methods, page 13, line 148–149: "(e.g., datapoints from rows 2-4 in Fig 2A)"—the rows in this table are not numbered, which makes the interpretation of this line more challenging than it needs to be (does the first row of the table count as zero, or does it count as one, or does the top row [describing the "NC" fold change] not count as a row, …). I recommend either (1) adding row numbers to the table in Figure 2A or (2) just removing this parenthetical entirely, since it doesn't add much.

28. Methods, page 13, line 149: Not necessary, but you may want to refer to Figure 4 when you mention interval estimates here—this is the earliest part in the paper that mentions interval estimates. (And, as I understand it, Figure 4C contains an example of an interval estimate that—in a plot in the style of Figure 2C—would "cross" the [-1, 1) region.)

29. Methods, page 13, lines 155–156: "We denote axis tick labels as x' to clarify that the fold change data is not changed with these reversed transforms."
    1. I don't think x' is used properly in this section. If (for a given axis tick), the corresponding Con-MFC value is x and the axis label (the FC aka MAD-FC value) is x', then shouldn't these functions be set up in terms of x? And then the equation given in Figure 2 under "Axis Tick Label Reassignment" would be

$$x' = f_M^{-1}\left(f_C^{-1}(x)\right)$$

    2. Also, I'm not sure that introducing the x' notation here is necessary. I don't think it improves readability much, and if anything it could confuse someone who is skimming the paper into thinking that you're taking the derivative of something.

30. Methods, pages 13–14, lines 156 and 158:
    1. The way the function f^{-1}_C (in LaTeX code) is written out here makes it hard to see the minus sign before 1—unless you zoom in very closely, it looks like f^{1}_C.
    2. Also, it looks like the f^{-1}_M function on line 158 is actually written out here with two dashes ("--1").
    3. I assume that at least the first of these problems will be fixed in typesetting (not sure about the "--1" issue—please fix that). However, I still suggest trying to fix these in the current version of the manuscript, if this is feasible.

31. Figure 2:
    1. Lines 127–128: "|FCZ| column denotes absolute value of fold change units from zero"

    1. The introduction of additional terminology here seems unnecessary—could the already-defined notion of fold change from no change be reused here?
    2. Also, as discussed earlier, I think “zero” is incorrect here and be should replaced with “the reference point”.

  2. The column names in Figure 2A are difficult if not impossible to decipher from the figure and caption alone (I understand that e.g. “MFC” is defined in line 146 of the Methods, but each Figure and caption should ideally be self-contained—also, it’s never exactly stated what “MFC” stands for [although I can guess that it’s “mirrored fold change”]). I recommend using more explicit column names and/or extending the caption to describe the meaning of these columns. If more space is needed in order to make this happen, maybe it would make sense to split Figure 2A into a stand-alone Table.

  3. I don’t think there’s a strong need to have MAD-FC values in Figure 2A: there’s never a situation where they don’t equal the original FC values, right? (If there is such a situation, I missed it!) So I propose just omitting that column (which should give you more space to spell out column names in more detail, as suggested above).

  4. The x-axis label in Figure 2C (“M-FC”) should be renamed to “MFC” to be consistent with Figure 2A and the rest of the text.

  5. This isn’t very important, but you don’t need to say “-FC” or “+FC” to indicate the directionality of fold changes in Figure 2A—you could just say “-” or “+”. (If anything, removing the unnecessary “FC” text here might make it possible to enlarge the font size of the “-” / “+” symbols, which would make the table easier to read without zooming in.)

  6. Line 134: “(D) The transforms in (C) and (D) are reversed...”—is this intentional? It is confusing to refer to (D) from within the description of (D). If you end up keeping Figures 2E, 2F, and 2G (see below), I suggest replacing the first (D) in this line with (E, F, G) or something.

  7. I don’t think Figures 2E, 2F, and 2G all need to exist; just one of them should be sufficient.

  8. See the comment above (“Methods, page 13, lines 155–156”) about the x’ notation—I think the equation given under “Axis Tick Label Reassignment” should be corrected.

32. Figure 3: Aside from the data issues discussed in “Major points”, I have some minor suggestions:

1. It looks like the y-axis labels of the volcano plots were created by using a minus sign character between “p” and “value”. I’m not sure how easy it is to change in your plotting software (if it’s challenging, feel free to ignore this), but I recommend using a smaller dash to just say “p-value” (which reduces the risk that anyone would misinterpret these labels as “p minus value”).

2. I propose removing the color legend in Figure 3F since it seems identical to the one in Figure 3C.

33. Figure 4: Even when viewed in high resolution, subfigures D, E, and F are difficult to read. It looks like the error bars have been cut off by the x-axis line? I strongly suggest either explaining this situation in the caption or enlarging these subfigures to make the entirety of the data visible.

34. Figures 4, 5, 6: What do the colors in subfigures A, B, C represent? If these colors were arbitrarily assigned, that’s fine—but please mention this in the captions.

35. Results, page 14, line 174: “to investigate mechanisms for their therapeutic effect”—please rephrase this so that the meaning of “their” is clearer (I assume it refers to the glucocorticoid(s)?, but the sentence as is is unclear).

36. Results, page 14, line 176: “summarize the trends” is vague and can probably be removed (maybe you could delete “to identify interesting genes and summarize the trends” in this sentence entirely—this phrase is unneeded since you later explain volcano and MA plots in more detail).

37. Results, page 14, lines 176–178: “Volcano plots are a scatterplot that visualizes statistical significance versus effect size and enables a quick identification of genes ...” → “Volcano plots are [scatterplots] that [visualize] statistical significance versus effect size and [enable] [the] quick identification of genes …” (fixes grammar issues)

38. Results, pages 14–15: “We produce volcano plots with log, linear, and MAD plots of fold change for this dataset (Fig 3 A-C).”
    1. I think the second “plots” should be replaced with “transforms” or something—the current wording implies “plots of plots”.

2. I recommend rephrasing this to just say “Figures 3A–C show volcano plots …”, rather than saying “we produce” (which goes without saying).

39. Results, page 15, lines 179–181: “While the log plot has symmetry and allows us to compare positive and negative fold changes, the magnitude of fold change datapoints are not proportional to their distance to the origin (Fig 3A).”

    1. The meaning of the term “origin” is unclear in a volcano plot. I recommend replacing it with something else (e.g. rephrasing the sentence to end with “… proportional to their distance on the x-axis to the reference point” or something).

    2. I don’t think “magnitude” is the best word, here, because it isn’t clear how a point in a scatterplot can have magnitude.

    3. Also, I believe there are some grammar issues here—focusing only on grammar, the current middle portion of this sentence could be reworded as “[…] the magnitude of each fold change datapoint is not proportional to its [...]”

    4. “allows” is also not an ideal word to use, for multiple reasons—“helps” or something similar would be better-suited. (See also line 181: “The linear plot allows us to…”, and line 183: “The MAD plot of fold change allows ...”)

    5. I don’t think citing Figure 3A at the end of this sentence is a good idea, since—if I’m understanding this correctly—the argument here is that the distance from each fold change value’s corresponding point to the reference point is not proportional to the “fold change from no change” value for each fold change. Since Figure 3A is just a scatterplot, the reader can’t see the exact underlying fold change values for each data point, so it’s hard to visually recognize this sort of bias here. I think instead you want to refer the reader to Figure 1E—maybe something like “While the log plot (Figure 3A) has symmetry […] are not proportional to their distance to the origin (Figure 1E).”

40. Results, page 18, line 271: “The MAD-FC visualization can also be useful for mapping fold change data to color gradients.”—I think you should replace “MAD-FC visualization” with “MAD-FC transform”, since you’re not really using the visualization itself as the input to the heatmap—you’re using the transformed data.

41. Results, page 18, line 272: “comparison of between heatmap of” seems like a typo.

42. Results, page 18, lines 273–274: “Visualizing fold change with a log mapping to color gradient […]”—This is worded confusingly. I think what you mean to say is that you are mapping the logarithmically transformed fold changes to a color gradient and visualizing that.

43. In Figure 1, the order of plots is linear then log then MAD. In Figures 3, 4, 5, 6, and 7, though, the order of plots is log then linear then MAD. I recommend making this consistent; maybe changing Figures 3–7 to go linear then log then MAD would be desirable, since this way the log and MAD plots will be next to each other (and hopefully easier to compare; the main "competition" MAD plots face are log plots).

44. In Figures 4, 5, and 6: Please indicate within the Figures that the top and bottom halves correspond to completely different datasets (and that the top datasets are simulated). This is not clear from the images alone.

45. Figure 7C: in the other plots using MAD-FC scales, it's made clear that the position of a point in the plot does not necessarily "match" the axis labels—e.g. in Figure 2D, the point located at (-2, 2) actually represents a fold-change (x-axis value) of 1/3. This is fine, but it's not immediately obvious how this "distortion" applies to non-spatial representations of MAD-FC-transformed data. To clarify—does Figure 7C assign colors based on the original fold changes, or based on the "Con-MFC" fold changes given in Figure 2A? (I am fairly certain that it's the latter, but I do not see this explicitly discussed anywhere in the text.)

46. Discussion, pages 19–20: "Different visualizations exhibit unique behaviors that highlight distinct aspects of the underlying data" (lines 283–284) and "Different visualization yields unique summaries of the data that will influence which datapoints are prioritized for further investigation" (lines 305–307) are redundant; I suggest removing one of them, or at least updating these sentences to not be so similar.

47. Discussion, page 19, line 287: "This limitation interferes with acquiring a holistic summary of fold change values."—"interferes with" is a bit awkward, so I recommend rephrasing this.

48. Discussion, page 19, line 289: "linear and proportional" seems redundant, since the rest of the paper uses "proportional" to mean "linearly proportional".

49. Discussion, page 20, lines 293–294: "While individual points can be read from a log plot, it is extremely difficult to identify trends from point clouds in a log scale."—This is a very strong claim that should be toned down. The term "trends" is very general; it's definitely possible to identify certain types of trends / patterns in scatterplots that use log scales, and in some cases log scales can even help users identify some types of trends that may be challenging to observe with linear scales.

50. Discussion, page 20, lines 294–295: "MAD-FC are designed to facilitate this process by preserving a linear and proportional encoding of fold change values."

1. Small typo: “MAD-FC are designed to” → “MAD-FC [plots] are designed to”
2. It isn’t clear what “this process” is—“identifying trends from point clouds”? I recommend being more direct here.
3. This sentence is also noticeably similar to another sentence earlier in this paragraph: “MAD-FC plots are designed to maintain readability, symmetry, and proportionality.”
4. In general, this paragraph is a bit awkward because it talks about log plots, then about MAD-FC plots, then about log plots (“While individual points can be read from a log plot, it is extremely difficult to identify trends from point clouds in a log scale.”), then about MAD-FC plots. Restructuring it should help readers.

51. Discussion, page 19, line 298: I recommend changing “as found with” to the more standard “as in”, but it’s not very important. (If you’d like to emphasize that you showed this distortion earlier in the paper, then do that explicitly here [since some people will read the paper out of order].)

52. Discussion, page 19, lines 299–300: “We enhance linear visualization of fold change with a transform strategy that is borrowed from log plots, where the datapoints and axis tick labels undergo different transformations.”—I might be missing something, but I don’t see how log plots play into this. A log plot is a log plot because it uses a logarithmic scale. As discussed earlier, the tick labels don’t actually matter that much.

53. Discussion, page 19, lines 300–302: “This produces a visualization that has a linear plot’s ease of interpretation combined with the ability to compare the magnitude of fold change across all measurements.”—This seems somewhat redundant with the paragraph above this one. I recommend trying to tighten up the language here.

54. Discussion, page 19, lines 304–305: “This new visualization may be a useful tool for gaining a more intuitive summarization of datapoint values in fold change datasets.”—This is technically correct, but could be simplified by replacing “gaining a more intuitive summarization of datapoint values in fold change datasets” with “more intuitively summarizing fold change values”.

55. Discussion, page 20, line 307:

56. Discussion, page 20, lines 307–309: “A fold change visualization can also influence the value of the thresholds use to determine if a datapoint is considered noteworthy or not, which can significantly alter the interpretation of fold change data (9).”
    1. Small typo: “thresholds use to” → “thresholds [used] to”

2. You don’t need to completely reword this sentence, but “significantly alter the interpretation” is almost an exact quote from the title of reference 9 (“Fold change and p-value cutoffs significantly alter microarray interpretations”). I strongly recommend rephrasing this in your own words.

57. Discussion, page 20: I recommend adjusting “comparing effect size across broadly related experiments (11–13)” to mention in-text that these references constitute work that the authors themselves have done in this area (i.e. that they are self-citations).
