# Supplementary material for: MAD-FC: A fold change visualization with readability, proportionality, and symmetry
Source: PLoS One. 2024 May 31;19(5):e0304632. doi: 10.1371/journal.pone.0304632 (PMC11142613; doi:10.1371/journal.pone.0304632)
Supplement: Attachment — Submitted filename: 202311_CommentsToAuthors.pdf [file pone.0304632.s003.pdf]

# Review 2 of Corliss *et al*., 2023 (“MAD-FC: a fold change visualization with readability, proportionality, and symmetry”)

The authors have done an excellent job improving the paper. The additional rigor and detail introduced in this revision, including the additions to Figure 1 and the new Figure 8, makes the paper much stronger—thank you!

In particular, the revisions made to the four desirable criteria of a fold change visualization (readability, proportionality, symmetry, and high dynamic range) make the foundation of the paper much clearer. I still have some lingering questions about the readability criterion, however, and I have a few ideas that I think would improve the presentation of the other three criteria.

The majority of the remaining suggestions I have now are minor points; many of these are debatable issues with phrasing or inessential suggestions, but there are some remaining clear errors that should be addressed.

In the comments below, I will refer to line numbers in the “clean” copy of the revised manuscript.

## Major points

1. **Definitions used in “Readability”:** The new definitions used in the context of readability help illustrate your intent much better—however, I still have a few remaining issues with these terms.
    1. The definition of readability given in the introduction (lines 60–69) seems to only describe this in terms of plots where the fold change is used as a spatial encoding, i.e. not including Figure 7 (which shows heatmaps of fold changes).
        1. You could probably define some sort of relationship between spatial and color-based encodings that allows us to apply “readability” to the latter, but the introduction section does not seem to do this as of writing.
        2. It’s fine if “readability” only applies to spatial plots (I think going very in-depth about how MAD-FC can be used for heatmaps is probably out of scope of this paper). However, I would at least recommend adding a small disclaimer (likely to the introduction) that the descriptions of these four properties are mainly given in the context of spatial plots.
        3. This also impacts the other three properties (proportionality, symmetry, and dynamic range) somewhat—however, these three properties seem a bit easier to describe in the context of a color-based encoding like that used in Figure 7. The possible more formal definitions of proportionality and symmetry I mentioned below (see “Introduction, lines 75–83 and 86–88”) may help with such a description? That being said, as stated above and below I do not think these particular issues are essential to resolve before publication.

    2. Distinguishing raw and labeled plots has made the explanation of readability much clearer, I think! The main issue I have with the current presentation is that the paper implies that these are two mutually exclusive options—but, as Figures 1D and 1E show, it is possible to have both types of tick labels in the same plot.

        1. Of course, in practice most plots (like Figures 3–8) will only show one type of label, so I think your distinction still makes sense. I will leave the exact way of handling it up to the authors, but I recommend at least adding a small disclaimer somewhere that there doesn't have to be a "dichotomy" between raw and labeled plots. (Unless I am missing something—or, if you feel that combining labels in this way makes the plot hard to read, then it would be worth discussing that in the text.)

        2. I think taking some more time in the introduction section that explains these terms (lines 107–109) to go into more detail would help clarify things.

2. **Evaluating proportionality and symmetry:** Introduction, lines 75–83 and 86–88: The descriptions of how to visually evaluate proportionality and symmetry have made this section much clearer—thank you! This is not an essential suggestion, so feel free to skip it if you'd like, but I propose more formally defining the ways in which you evaluate these properties.

    1. For example, rather than saying that "Symmetry of a transform can be visually assessed by measuring the distance between synthetically generated pairs of fold changes of opposite direction with the same magnitude", it would be more correct to provide a mathematical definition of symmetry (e.g. given a transform function $f(x)$, this transform exhibits symmetry if $f(x) = -f(1/x)$).

        1. This accounts for the unlikely case where a transform is only partially symmetric for some reason (e.g. only up to within a certain distance from the point of no change)—such a transform should not be called "symmetric," and yet the visual assessment method currently described in the paper would result in this transform being called "symmetric" unless the synthetic dataset generated for testing extends far enough.

    2. Similarly, instead of describing the process of drawing a line between the largest positive fold change and the point of no change and then checking to see if all positive points fall on this line, you could say that a transform function $f(x)$ is proportional if, for all fold changes $x > 1$, $f(x) = mf_U(x) – mf_U(1) + f(1)$ given some real $m > 0$.

        1. At least, I think this correctly describes this relationship—this is based on the idea that $m$, which is the slope of the line passing through the point of no change in Figures 1C–1E (i.e. $(f_U(1), f(1))$) and an arbitrary positive fold change (i.e. $(f_U(x), f(x))$, should be equal to the slope of the analogous line for all other positive fold changes. There might be a more elegant way to formulate this, though.

    3. As mentioned, I don't think you need to make these changes—the current descriptions (using visual evaluation methods) are sufficient. However, being more exact here would help future researchers evaluate their transformations on the same criteria with less work,

and it would open the possibility for objectively quantifying how well a transform adheres to these criteria.

    1. (It might also make it easier to adapt proportionality, symmetry, and dynamic range to color-based encodings like Figure 7—if you're only dealing with numbers rather than plots, it should be easier to apply these properties to color gradients.)

  4. If you choose to not make these changes (which is fine), I propose just adding a short note somewhere in the introduction (maybe alongside the proportionality and symmetry definitions) mentioning that the visual evaluation methods are slightly informal ways of testing these properties.

3. **Explaining "Dynamic Range":** Similarly, the new information about dynamic range (including Figure 1B) helps explain things a lot. Thank you!

    1. It took me a while to understand the new changes, but I think I understand the point being made in the "Dynamic Range" section of the introduction now. To help other readers see your intent clearly, I have two suggestions:

        1. Maybe explain more directly (in the "Dynamic Range" section of the introduction?) that, when visualizing data spanning many orders of magnitude, linear scales make it harder to distinguish small values—i.e. that these scales are dwarfed by "outliers." This point is already made in the "Dynamic Range" section of the introduction, but (if this seems reasonable) I recommend going into more detail in the text about why exactly we see the effect in Figure 1B.

        2. The sentences in the introduction explaining why linear and MAD plots have only medium dynamic range should be expanded in order to clarify this. Maybe refer to Figure 1B and/or Figure 8 from lines 117–118 and line 136? When I first read line 136, I was confused as to why MAD plots had only medium dynamic range; seeing Figure 8 helped everything click.

## Minor points

1. Abstract, line 35: This is an extremely minor point, but you use the term "MAD-FC plot" here (despite only defining the term "MAD plot" later in the paper). There is thus a very slight inconsistency.

    1. You could probably leave this as is (I do not think it is essential to fix), but maybe this sentence could be rewritten to be consistent with the rest of the paper—perhaps something like "We argue that MAD-FC transformed fold changes may yield more useful visualizations than log or linear transformed fold changes […]"? But the decision is the author's call—whatever you think would be best.

2. Introduction, line 44–45: "Typically, scientists visualize fold change with a plot using a log or a linear transform, the latter of which presents raw fold change values (2)."

    1. "with a plot" could probably be removed from this sentence, since I believe it goes without saying (very minor, though, so feel free to disregard).
    2. The term "linear transform" might confuse some readers—if it wouldn't be too much trouble, I would recommend just removing the four uses of "linear transform" throughout the paper (since when you visualize the raw fold changes you're not really transforming the data at all).
        1. As a less dramatic alternative, maybe you could add a "simply" to the end of this sentence, i.e. "simply presents raw fold change values", or otherwise rephrase it a bit to be extra clear that your use of "linear transform" just amounts to visualizing the raw fold changes.
    3. A slightly more important issue: to my understanding, the reference to (2) here (Love et al. 2014, the DESeq2 paper) supports the first claim in this sentence (that people typically visualize log-transformed fold changes), but that paper doesn't provide any examples of visualizing raw / linear-transformed fold changes. Unless I am missing something, I recommend moving the reference to (2) earlier in this sentence to be after you mention log fold changes, just to be clearer that (2) is only a reference regarding log fold changes.
        1. If you know of any examples of papers that visualize raw fold changes, you could cite those here after citing Love et al. 2014—I think reference (5) (O'Shea et al. 2017, the source of the data in Figure 4D–F) should be sufficient. (Before I realized that (5) would be a good example of a paper that visualizes raw fold-changes, I dug up another example—Figure 4 of https://onlinelibrary.wiley.com/doi/full/10.1111/eva.13142.)

3. Introduction, line 45: minor phrasing issue—I suggest adjusting "Both transforms have a unique set of properties" to something that indicates that these transforms (or scales, see above point) do not have the exact same set of properties (which seems to be what the current sentence implies, although it's clear to me what you mean).
    1. I think the easiest way to fix this would just be adjusting the sentence's start to say that "Each transform has" rather than "Both transforms have," if this seems reasonable.

4. Introduction, line 56: The changes in this section are great; thank you! The only tiny comment I have is that you may want to adjust this line to say "With this encoding, [the raw] fold changes of […]", rather than just "With this encoding, fold changes of […]". This will make it unambiguously clear to the reader that 2, 1/2, 3, and 1/3 are raw / linear fold changes, rather than log- or MAD-FC-transformed fold changes.
    1. This could also apply to line 58, I believe.

5. Introduction, line 62: minor grammar issue—I think you mean to say "values" and "locations" to match the plural tense of "visualized datapoints". I bring this up because the use of the

singular-tense words “value” / “location” could confuse readers.

6. Introduction, line 66: the term “raw logarithmic axis tick labels” has not yet been defined, and the meaning is not immediately obvious without reading other parts of the paper (e.g. Figure 1D). I suggest either (1) briefly defining this term, (2) rewriting this sentence in a clearer way, or (3) referring to one of your figures to explain this term.
    1. Option (3) might be the best; maybe you could say something like “[…] raw logarithmic axis tick labels (e.g. rightmost y-axis labels of Figure 1D) […]”?
    2. This also applies to line 107 of the introduction.

7. Introduction, line 73: Minor grammatical error (“magnitude” → “magnitudes”), I think.

8. Introduction, line 74: I assume “transformed units” here refers to the linear / log / MAD-FC transformed fold changes; this is fine as is, but if feasible you may want to make it a bit clearer (since the term “transformed units” has not been defined yet and is a bit vague).
    1. Maybe something like “[…] between the transformed fold changes and corresponding fold change units […]”? This could still be made clearer, but I think it would help.

9. Introduction, line 78: Minor, but I’m not sure “trend line” is the correct term here—I think you could just say “line”, unless I’m misunderstanding something.

10. Introduction, lines 98–99: “[…] a linear scale can typically capture 8 orders of magnitude on a log2 scale, or about 2.5 orders of magnitude on a log10 scale.”
    1. The notion of “a linear scale … on a log scale” seems confusing to me. I think your intent is to say that a typically-sized plot can span about 2^8 or 10^2.5 units? I think saying “units” or (like in the abstract) “log2 space” would help clarify this.
    2. Also: it’s a small point, but these numbers (8 units in log2 space, 2.5 units in log10 space) are repeated a couple of times throughout the paper (in the abstract, here in the introduction, in the results near figure 8, and in the discussion).
        1. This is fine as is, but I propose just giving these estimates once (probably here in the introduction) and otherwise just referring to the idea of “multiple orders of magnitude” or “data requiring a high dynamic range” (e.g. as in the abstract on line 36) or something. I think this will convey the same idea while being a bit less confusing to the reader.
        2. (But this is completely optional—you could make an argument that repeating these estimated limits throughout the paper is a good thing, so that people skimming the paper

will probably see them. So please feel free to disregard this comment.)

11. Introduction, lines 102–103: Thank you for addressing that comment! I have just a slight issue with this new sentence, sorry—I think it would be more correct (or at least applicable) to say something like "[…] there are instances where two datapoints are located closer together in [a log plot than a corresponding linear plot] and therefore harder to distinguish.", rather than framing this in terms of log space / linear space.
    1. Since $\log(x) < x$ for $x > 0$, I think most (all?) arbitrary pairs of values ($x_1$, $x_2$) will be located closer together in log space than in linear space—but this is secondary to the purpose of this claim, which is from a visualization perspective.

12. Introduction, line 114: I think this should point to Figure 1C instead of 1B.

13. Introduction, line 116: This is fine as it is, but using "compressed between" for both of these intervals (with very different sizes) seems a bit confusing. Maybe you could say "span" for the second interval ($(1, \infty)$)?

14. Introduction, line 116: Similar issue to the above one, but less important—the paper uses the word "between" with interval notation in a couple of places. This is fine, but (at least to my understanding) "between" usually refers to the set of numbers within a range, not necessarily including the bounds of that range—so I think avoiding the use of it in this context makes sense. If it isn't too much of a pain, I propose replacing uses of "between" in the context of interval notation with "in the range" or "in the interval" or something like that. (But only if this is not too much of a pain—this is not an essential thing to fix at all.)

15. Introduction, lines 119 and 130: I recommend being more specific with which set of y-axis labels you are referring to. Based on the caption of Figure 1, it looks like both cases the "raw" plots are the inner (right) set of y-axis labels, and the "labeled" plots are the outer (left) set of y-axis labels—but this is not clear to a first-time reader. (I think referring to Figures 1D / 1E in the same sentence as when you introduce raw log / MAD plots will confuse some readers into thinking that these figures only show raw versions of these plots.)
    1. Similarly, I recommend adding to the references to "labeled" log / MAD plots (lines 123 and 133) to point to the corresponding outer y-axis labels in Figures 1D and 1E. Eventually a reader would consult the legend of Figure 1 and figure this out, but I think being extra clear here will help avoid this confusion.

16. Introduction, line 125: I recommend extending the parenthetical of "(back-transformed tick marks are not linearly mapped to the axis scale)" to make it clearer that this happens because

the underlying scale is itself not linear.

17. Introduction, line 129: Unless I'm misunderstanding something, I think this first sentence should be adjusted—the use of "raw" here implies that the "stretching out" of the fold changes in a MAD plot is due to the plot being a raw plot, not due to it being a MAD plot. The meaning is decipherable from the current text, but it is a bit unclear at first.
    1. I think it would make sense to remove the "raw" from the first sentence, then maybe briefly mention in the next sentence what the units of a "raw" MAD plot are (MAD-FC transformed fold changes; you can refer interested readers to the Methods section here for details)

18. Introduction, line 133: Grammatical error ("a labeled MAD plot has readability because it uses extraction of datapoints with linear interpolation between axis tick marks")—I think this should be rewritten to explain why a user would find it easy to "extract" datapoints from a MAD plot.

19. Methods, line 157: Minor grammatical error (comma splice—you could replace it with a semicolon (;)).

20. Methods, lines 160–161: The sentence "We start by defining symmetry between positive and negative fold changes." can probably be removed, I think—in light of the revisions to this section, this sentence does not add much.
    1. The sentence before it, "To obtain symmetry, […]" could also be removed—but I will leave that decision up to the authors. As an alternative to removing that sentence, you may want to replace "To obtain symmetry" with "In order for a plot of fold changes to be symmetric" or something—this would work as a clearer transition to the next paragraph (introducing the mirror transform).
    2. But these are minor stylistic points—feel free to leave the text as is if you prefer.

21. Methods, line 193: The use of "[…] but if we wish to produce a MAD plot […]" here implies that the plot shown in Figure 2D is not a MAD plot—although I think, using the terminology introduced earlier, Figure 2D counts as a "raw" MAD plot. In either case, I suggest adjusting this text to clarify.
    1. A similar issue occurs in the note at the bottom of Figure 2, which states that "E is for MAD plot only" (implying the same thing, I think—that Figure 2D is not a MAD plot).

22. Data and material availability, line 209: I don't think (4) (which points to "Marc C. org.Hs.eg.db: Genome wide annotation for Human. 2019.") is the correct reference for Bioconductor; I think https://genomebiology.biomedcentral.com/articles/10.1186/gb-2004-5-10-

r80 would be the correct reference?

23. Data and material availability, line 209 and row 1 of Table 1: minor typo, “DSeq2” → “DESeq2”

24. Data and material availability, line 209 and Figure 3 caption, line 260: minor typo, “Walde” → “Wald”

25. Data and material availability, line 211: “Figure 6C from the reference publication (5) […]”
    1. Thank you for explaining the provenance of this data! I consulted reference 5 (O’Shea et al. 2017), and it does not look like Figure 6 there has a panel named “C”—it just has two subplots (and I think you are just using data from the left subplot, for HCC1954-P). Please correct this sentence (e.g. saying “Figure 6 (left)” or something).

26. Data and material availability, lines 217–218: The text “label free quantification for ubiquitin interactors of different linear ubiquitin lengths” seems to be exactly copied from the description given at https://rdrr.io/bioc/DEP/man/UbiLength.html; please rewrite or remove this text.

27. Results, line 232: very minor point, but I think “across” would be a better word to use here than “with”—you’re not comparing the plots with the datasets, but you are comparing the plots with each other across different datasets.

28. Results, line 231–233: if feasible, I recommend adding to this sentence to mention that you’re also considering different types of visualizations (volcano plots, box plots, violin plots, etc)—this is the second major distinguishing factor across Figures 3–7.

29. Results, line 235: I don’t think all of the genes measured in this experiment were differentially expressed, so saying “an RNA-Seq dataset of differentially expressed genes” seems incorrect—I recommend rephrasing this.

30. Results, line 242: “The linear plot facilitates comparing the magnitude between positive fold change datapoints”—I recommend replacing “magnitude between” with “magnitudes of” to make this sentence clearer. I initially misread this and thought it was talking about comparing the magnitudes between positive and negative fold changes.

31. Results, line 269: minor, but I think you can remove the “it’s” from this line.

32. Results, line 270: I think “quantile” should be changed to “quartile” here, unless I am misunderstanding something.

    1. This also applies to other uses of quantile (in line 313 in the Figure 5 caption and in line 379 in the discussion), I believe.

33. Results, lines 270–273; lines 299–301; and lines 320–321: To my understanding, the simulated dataset used in Figures 4, 5 and 6 is the same dataset (maybe with slight modifications between figures)—but the descriptions given here do not make this clear. I recommend shortening the latter two descriptions in favor of just explicitly saying that this is the same or a similar simulated dataset as used in Figure 4, rather than adding an extra explanation for every figure.

34. Results, line 287–288: I think this is the one remaining use of “reference point of no change” in the revised paper. As discussed in the previous round of reviews, I suggest removing the word “reference” to maintain consistency (since the term “point of no change” has now been defined, but the term “reference point of no change” has not)—but this is a very minor issue now.

35. Results, line 307: I think the reference here should point to Figures 5D–F, rather than just Figure 5.

36. Results, line 308: minor grammar issue: “compresses” → “compress”

37. Results, lines 352–353: “Although most bioinformatics datasets satisfy this condition, there are exceptions.” I think you mean to say “do not satisfy” here?

38. Discussion, lines 373–375: I agree with the softened version of this sentence more than before —thank you!

    1. However, I feel that “it is extremely difficult to identify linear trends from point clouds in a log scale” is still a bit overzealous as a general claim. Going off the statement earlier in the sentence that “individual points can be read from a log plot with those familiar with the log scale spacing of values between axis tick labels,” there is not a reason why identifying linear trends for these same people would be extremely difficult—the term “linear trends” is also sufficiently general that I would be very wary of saying this as a general fact.
    2. For the sake of being as accurate, as possible my vote is just for removing the “extremely” in this sentence—but if you feel strongly that it should be kept, then I think that is fine. An alternative would be just indicating that it is your opinion (“we contend that it is extremely difficult to […]”)?

39. Figure 1:
    1. Caption, line 138 (header): The grammar here seems incorrect. I think you mean to say something like “Evaluating linear, log, and MAD plots on our four fold change visualization properties”?
    2. Caption, lines 142–143: It is a bit ambiguous what the “legend” referred to here is, but I assume that it is the table of fold changes vs. fold change units. Maybe it would be worth labeling this table to make this clearer? (But if this would be inconvenient, it’s okay to leave it as is.)
    3. Caption, line 143: “(B) log” should say “(D) log”
    4. Caption, line 147: very minor grammar issue (“mapped in” should be changed to “mapped to”, I think).

40. Figure 2: The new version of this figure looks much nicer; thank you! I only have minor feedback here.
    1. Caption, line 178: extra closing parenthesis “)” given.
    2. I’m not sure that the new table of “Axis Notation Formats” is necessary to include. I understand that these three formats are implemented as an option in the current MAD-FC code (https://github.com/bacorliss/mirrored_axis_distortion/blob/b536c56f067a499fd44bf4160ed4e8ec635b0978/R/mirrored_axis_distortion.R#L123-L124), but I am not sure that this is important to show in the figure or caption.
        1. The fraction vs. decimal vs. exponent choice is also mentioned in lines 199–200 in the methods section—I propose adding to the text there (explaining that this is an option in the R code), and removing the details about these options from Figure 2 and its caption. This isn’t an essential change, but I do not think having this information in Figure 2 will help readers understand the figure.
        2. If you’d like to keep the Axis Notation Formats table, I recommend adding another arrow to connect it with the x-axis labels in Figure 2E—this will make the purpose of the table clearer.
    3. See the comment above labeled “Methods, line 193” for a question about the note in this figure (“MAD-FC transform is B-D; E is for MAD plot only”).
        1. On a related topic, I think this note could be removed entirely (or merged into the caption)—removing the note is not a necessary change, but I think you could convey the same information more intuitively by adding a box outline surrounding panels B–D labeled “MAD-FC transform”.

41. Figure 3:

1. The text in lines 208–210 is helpful for adding context to this figure, but it does not explain what the samples represent or what sort of differential expression analysis was performed—the figure's caption also does not include this information. This should be addressed so that a reader can understand this figure without reading reference (3) and this paper's source code.
2. There is some more context given about this in the results section (lines 235–237), but the details of the differential expression analysis are still omitted.
3. Based on reference (3) and the provided source code (thank you!), https://github.com/bacorliss/mirrored_axis_distortion/blob/b536c56f067a499fd44bf4160ed4e8ec635b0978/mirror_fc/F3A_volcano_plot2.R#L47C1, it looks like the design formula used was "cell + dex", which I am guessing means that DESeq2 took into account information about (1) the cell line types and (2) treatment with dexamethasone. But I am not sure about this.
4. I apologize for being strict about this; I understand that this analysis is not the focus of this paper, but it does impact the provenance in Figure 3, and should thus be at least briefly mentioned in some detail.

42. Figure 4:

    1. It looks like the labels given on the x-axis are slightly incorrect, relative to the original version of this figure given in O'Shea et al. 2017:
        1. The fourth-from-the-left label, "EGFR", was changed to "EDFR"
        2. "MAPK 1/2 (T202/Y204)" was changed to "MAPK1/2(S217/221)"
        3. Less important: Some of the labels are missing spaces that were present in the original figure (e.g. "p70 S6K (T389)" → "p70S6K(T389)". If it's easy to do, I recommend adding back these spaces (they are probably not needed for making these labels understandable, especially since you are referring to the original publication of this data, but they would be nice to have).
        4. Much less important: the positions of "p70 S6K1" and "p70 S6K (T389)" were switched from the original figure (but this is not a big problem, since the data points shown were also switched).
        5. I am not sure if these issues are an artifact of WebPlotDigitizer or of manual data entry—in any case, please fix the two incorrect labels, and ideally also the missing spaces in the other labels. Thank you!

43. Figure 7:

    1. This is an extremely minor issue (and this might be fixed by the journal during typesetting), but I think all of the Ubiquitin names at the bottom of the figure should be capitalized

consistently—the name of hexa-ubiquitin is given in lowercase (“ubi6”) while the others are given in title case (“Ubi1”, “Ubi4”). Since the caption explicitly refers to “Ubi6”, I suggest updating the third columns of subfigures A, B, and C to also say “Ubi6” (if this is easy to do).

2. The extra information given in the caption helps explain this figure much more clearly—thank you! It is very useful to know that only every 10th protein is labeled on the y-axis. However, it is very difficult right now for a reader to see which of the thin rows each label refers to.
    1. My best guess right now is that the “ACAT2” label refers to the bottom row of each figure, and that the other protein labels occur in increments of 10 above this bottom row. However, this is not clear.
    2. I propose either (1) extending the length of the ticks corresponding to the “labeled proteins” to make these labels more readable (or doing something similar, e.g. coloring these labeled proteins’ ticks a different color), or (2) removing the protein labels entirely. This is because the protein labels are currently ambiguous. (I’m aware that the purpose of this figure is not to actually provide insights about this data, but to show that MAD-FC is applicable to these sorts of heatmaps of fold changes—removing the protein labels would thus help emphasize the important parts of this figure, i.e. the colored bars.)
3. This is not an essential change (and I imagine it could be a pain to implement, so please feel free to disregard it), but it may be helpful to sort the rows of proteins in this figure somehow (e.g. from lowest to highest average fold change across the three ubiquitins, assuming that this would impose the same row order for figures A, B, and C). This could make it easier to interpret coloring differences between the linear, log2, and MAD fold changes.
    1. (If there is already a significance to the ordering of proteins in this figure, then please feel free to keep it—although I would recommend explaining this ordering in the caption if so.)
4. This is the least important of all of these issues, but the color legends shown at the top of this figure have an extra “bar” jutting out horizontally of the middle of the linear and log2 legends—this bar is absent from the MAD legend. (See below for two zoomed-in screenshots; this artifact is only present in the log2 legend.)
    1. This isn’t causing any problems or anything, but I bring it up in case it is the result of a software bug or some visual artifact. If it’s not possible to remove these extra bars, it’s not a problem.

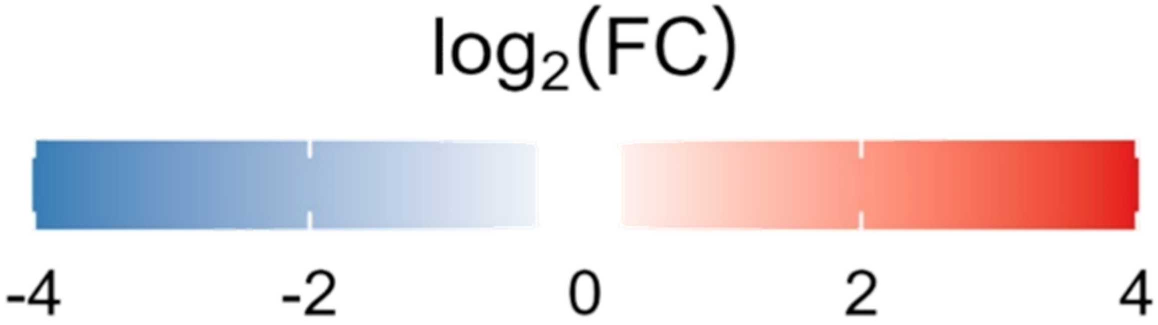


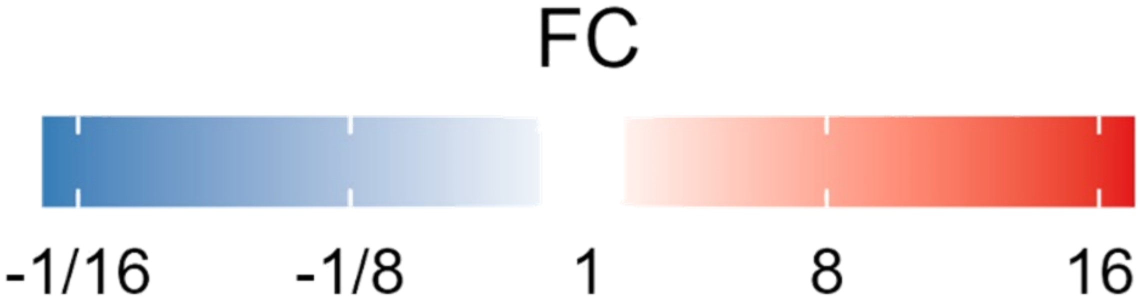


44. Figure 8: This figure does a great job of demonstrating a limitation of MAD-FC—thank you for including it. The only minor comment I have is that, if easy to do, it would be nice to have a legend included within the figure (although since the colors are already explained in the caption this is not an essential issue to fix).
